# A Rare Case of Sarcoidosis Presenting With Endobronchial Mass

**DOI:** 10.7759/cureus.98768

**Published:** 2025-12-08

**Authors:** Ramachandana G, Pankti Sheth, Viswesvaran Balasubramanian, Kona Lakshmi Chermisha Naidu, Ananya Reddy Aerra

**Affiliations:** 1 Interventional Pulmonology, Yashoda Hospitals, Hyderabad, IND; 2 Medicine, Gandhi Medical College, Hyderabad, IND

**Keywords:** endobronchial mass, granulomatous inflammation, mediastinal lymphadenopathy, pet ct scan, sarcoidosis

## Abstract

We report a rare case of sarcoidosis presenting with endobronchial mass in a 34-year-old male patient who presented with progressive shortness of breath and non-productive cough for one month. It is diagnostically challenging due to its non-specific presentations, as mass lesions can mimic malignancy. In this case, CT chest initially revealed mediastinal lymphadenopathy, multiple small peripheral lung nodules and mild right sided pleural effusion. Positron emission tomography (PET) CT revealed mediastinal lymphadenopathy and bilateral subpleural nodules*.* Bronchoscopy revealed a polypoid mass with a broad base in segmental bronchus of the right lower lobe. Histopathological examination of biopsy revealed granulomatous inflammation with lymphoplasmacytic infiltration, with no evidence of necrosis or malignancy. The endobronchial mass was removed using electrosurgical snaring with cryoextraction. Microbiological and cytology examinations were unremarkable. The patient was diagnosed with sarcoidosis and was initiated on oral prednisolone. This case highlights the importance of considering sarcoidosis in the differential diagnosis to ensure timely and appropriate management.

## Introduction

Sarcoidosis is a chronic, multisystemic granulomatous disease of unknown etiology that predominantly affects the lungs and characterized by the non-caseating granuloma formation in affected organs [1]. A characteristic pathology and the elimination of various infectious, interstitial and neoplastic illnesses serve as the basis for the diagnosis. Cough, dyspnea, fatigue, unintentional weight loss and night sweats are some of the symptoms observed in pulmonary sarcoidosis patients [2]. Clinical manifestations of sarcoidosis may vary according to the organ involved, ranging from asymptomatic to fatal presentations [3]. Atypical radiographic presentations of pulmonary sarcoidosis can be diagnostically challenging as the associated symptoms often resemble malignancy, particularly when rare mass lesions are present [4]. Although pulmonary involvement in sarcoidosis is common, endobronchial mass remains as a rare manifestation and it mimics malignancy [1].

We report a rare case of sarcoidosis presenting with endobronchial mass highlighting the need to consider broad differential diagnosis and comprehensive evaluation.

## Case presentation

A 34-year-old male patient, non-smoker, presented with progressive shortness of breath and non-productive cough for the past one month. Patient denied symptoms of fever, hemoptysis, chest pain and pedal edema. There was no history of weight loss, appetite loss, or symptoms suggestive of connective tissue disease or vasculitis. His past medical and surgical history were unremarkable.

**Figure 1:**
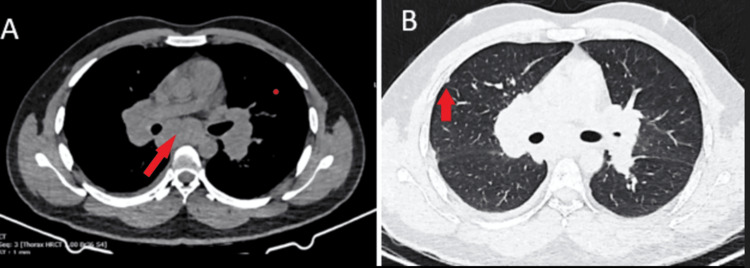
(A-B) High resolution CT chest – mediastinal lymphadenopathy with subpleural nodules The arrow in 1A points to subcarinal LN; the arrow in 1B points to subpleural nodule. LN: Lymph node

He was initially evaluated at another center, where a high-resolution CT chest revealed mediastinal lymphadenopathy, multiple small peripheral lung nodules with mild right sided pleural effusion with the arrows of 1A pointing to subcarinal lymph node (LN) and 1B to subpleural nodule (Figure 1). On presentation, his vital signs were as follows: pulse rate 100 beats/min, blood pressure 120/70 mmHg, respiratory rate 20 breaths/min and SpO_2_ 97% on room air. Respiratory system examination was unremarkable. Other systemic examinations were within normal limits.

Routine blood investigations, including complete blood counts, liver functions tests and renal function tests were within normal limits. Tuberculosis (TB) quantiferon GOLD test was negative. Other tests that excluded TB in our case include bronchoalveolar lavage acid fast bacilli culture and sensitivity (BAL AFB c/s), BAL AFB stain, BAL Gene Xpert, endobronchial ultrasound transbronchial needle aspiration (EBUS TBNA) AFB, EBUS TBNA Gene Xpert and EBUS TBNA AFB c/s. Serum Calcium (9.3 mg/dL) and Serum Angiotensin converting enzyme (58.9 units/L) levels were within normal limits. Antineutrophil cytoplasmic antibody (ANCA) serology was also negative. A whole-body positron emission tomography (PET) CT revealed fludeoxyglycose (FDG) avid (standardized uptake value (SUV) 18.3) mediastinal lymphadenopathy (3A, 4R, 4L, 7, 8, 10 R, 10 L) along with bilateral FDG avid (SUV 14.8) subpleural nodules. The subcarinal LN size observed was 32mm X 27 mm. Enlarged bilateral paratracheal, prevascular, subcarinal, hilar and paraesophageal LNs were observed.

**Figure 2:**
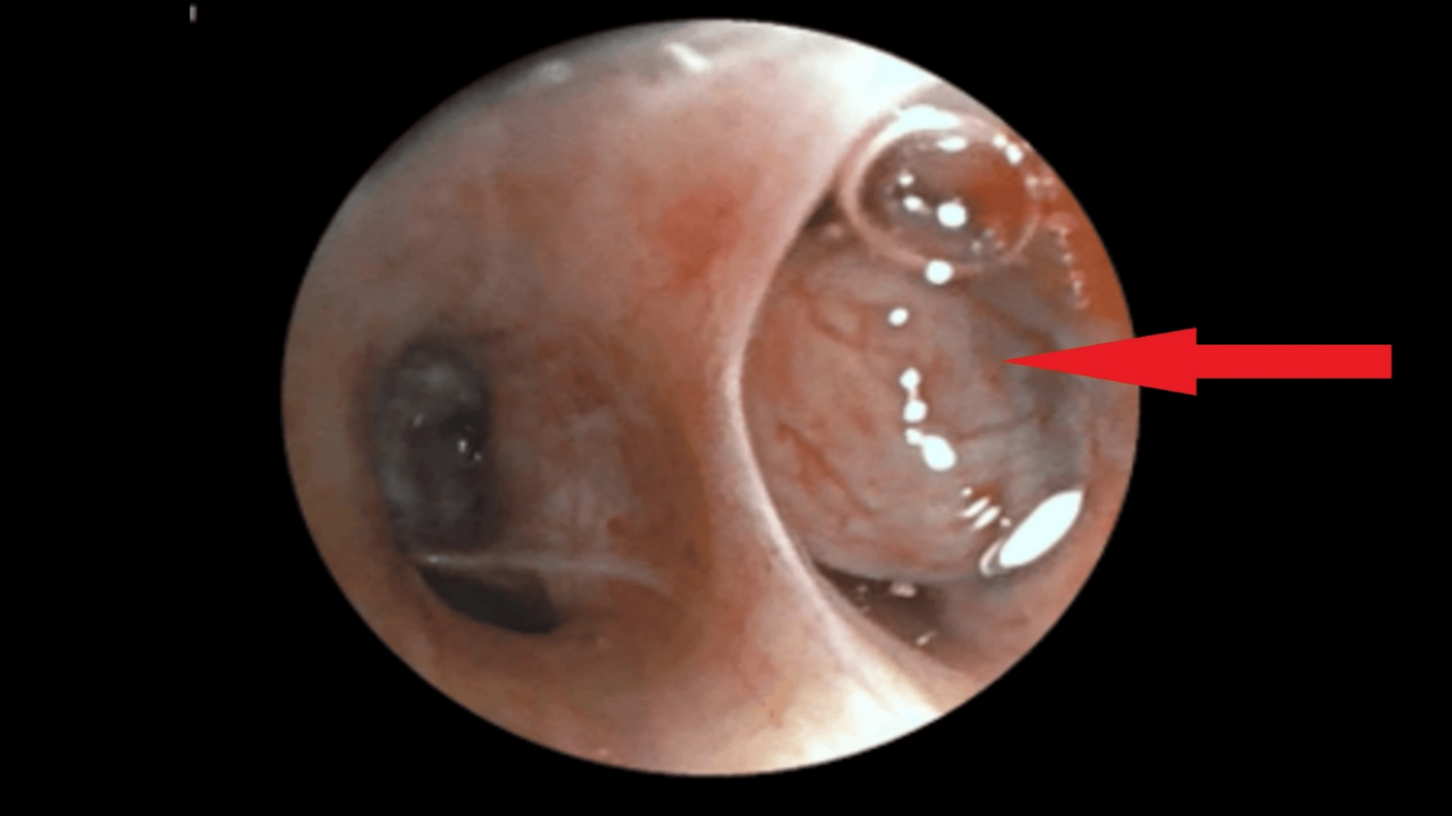
Endobronchial mass in right lower lobe (arrow) through laryngeal mask airway

Under general anesthesia, video-bronchoscopy through laryngeal mask airway revealed a polypoid mass lesion with a broad base in segmental bronchus of right lower lobe with the arrow pointing to endobronchial mass in right lower lobe (Figure 2). 

**Figure 3:**
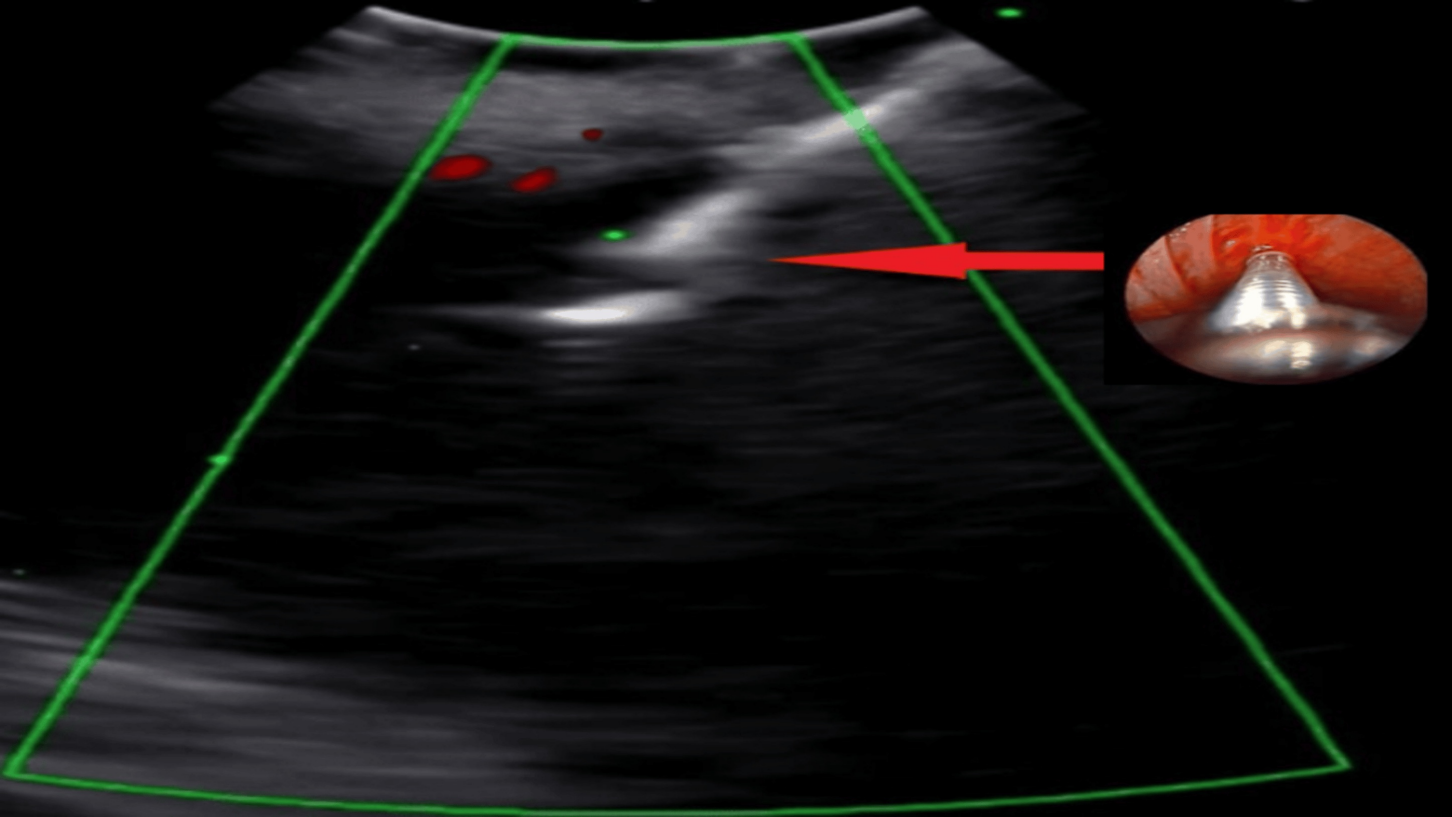
Linear EBUS TBNA from subcarinal LN The arrow points to endobronchial needle. EBUS: Endobronchial ultrasound; TBNA: Transbronchial needle aspiration; LN: Lymph node

Using a linear EBUS, TBNA and intranodal cryo-biopsy were obtained from subcarinal LN with arrow pointing to EBUS needle (Figure 3). After rigid bronchoscope intubation, electrosurgical snaring with cryoextraction of the endobronchial mass was performed.

**Figure 4:**
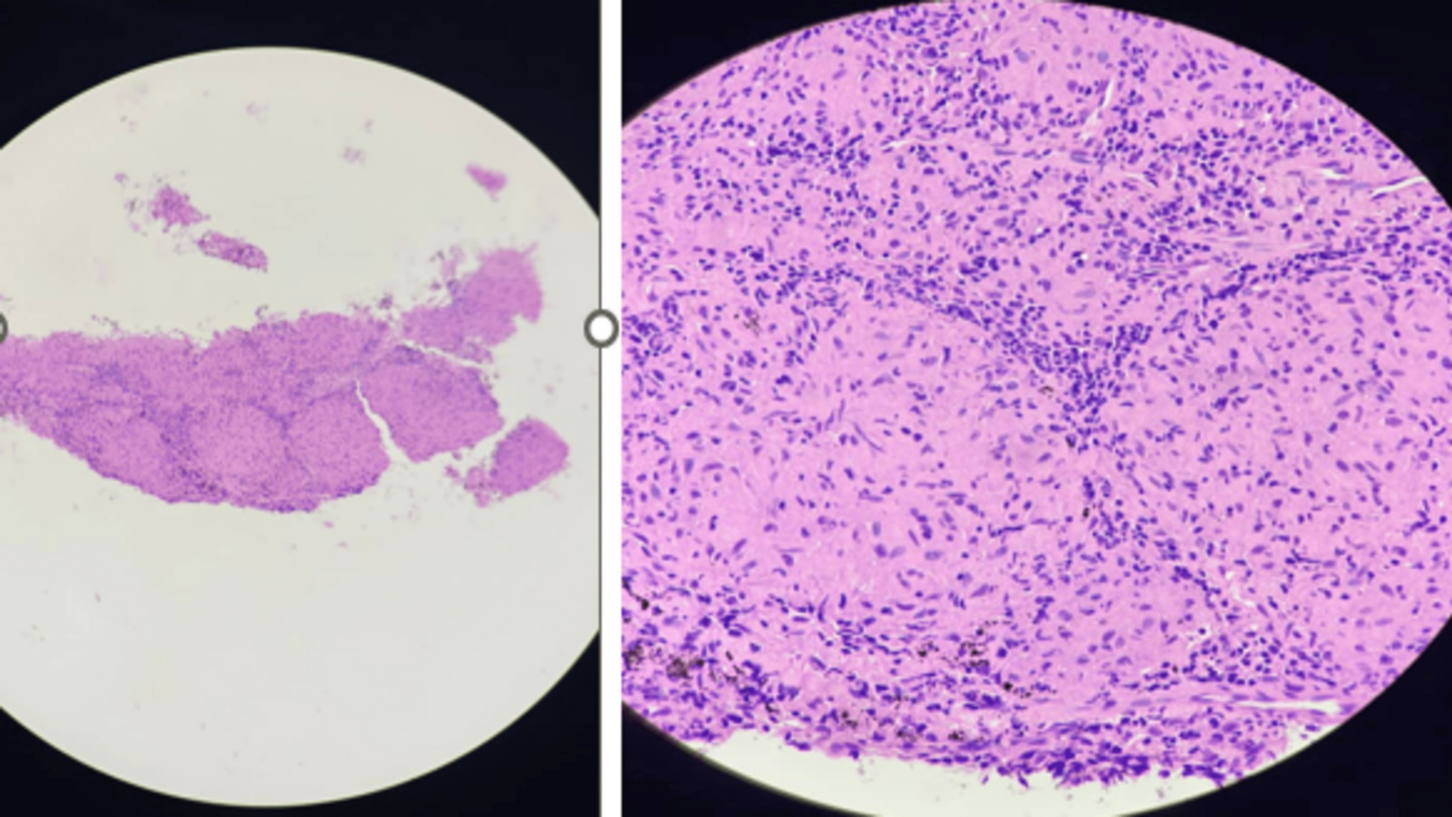
Histological findings leading to the diagnosis of sarcoidosis

Histopathological examination of biopsy of subcarinal LN and endobronchial mass revealed granulomatous inflammation with lymphoplasmacytic infiltration without any evidence of necrosis/malignancy (Figure 4).

Microbiological examination of BAL from right lower lobe and transbronchial needle aspirate were unremarkable. Cytology of BAL and TBNA were negative for malignancy. A diagnosis of sarcoidosis was established, and patient was initiated on oral prednisolone (0.5 mg/kg) once daily. Patient was asymptomatic at follow up visits for the last five months and radiological improvement was observed with a reduction in size of L1.

## Discussion

Sarcoidosis is a multisystem granulomatous disease of unknown etiology. It is characterized by non-caseating granulomatous inflammation, typically presenting in young and middle-aged adults. Diagnosis is based on clinical and radiological suspicion, histopathological evidence of non-caseating granulomas and exclusion of other granulomatous diseases such as TB, fungal infection and hypersensitivity pneumonitis [4]. 

While sarcoidosis commonly involves the respiratory system, multisystem involvement is characteristic of the disease [5]. Endobronchial involvement is common in sarcoidosis with Torrington et al. reporting airway abnormalities in 50-55 % of patients. Bronchoscopic findings range from mucosal erythema, edema, plaques, cobblestoning and nodules to hypertrophic tumor like masses and bronchial stenosis [6]. Endobronchial sarcoidosis presenting as a mass lesion is a less frequent but a challenging manifestation and has been rarely reported [7].

The proposed pathogenesis of sarcoidosis involves and exaggerated immune response to an unknown antigen, leading to the activation and accumulation of T-Lymphocytes, especially CD4+ T cells, macrophages and granuloma formation in affected tissues [8]. The exact pathogenesis of formation of endobronchial lesion in sarcoidosis in unknown, probably coalescence of granulomas gives the appearance of a mass [9]. The symptoms of sarcoidosis with an endobronchial mass are often non-specific, with patients presenting with chronic non-productive cough and progressive dyspnea with/without wheezing. Endobronchial involvement in sarcoidosis appears to be a risk factor for progressive disease and higher risk of extrapulmonary organ involvement [10].

Contrast-enhanced CT thorax is a valuable imaging modality for evaluating lymphadenopathy and parenchymal involvement. Classic parenchymal findings include small peri lymphatic nodules particularly in upper and mid zones, these nodules at times may conglomerate to form mass like lesions. 15-25 % of patients demonstrate atypical features such as ground glass opacities, necrotizing consolidations and reticulations with septal thickening [11]. In cases of endobronchial sarcoidosis, CT thorax may reveal reduced airway luminal diameter from endobronchial granulomas, extrinsic compression of the airways due to lymphadenopathy, bronchial distortion and mural thickening; however, its utility in evaluating airway patency is limited [12].

Sarcoidosis presenting as endobronchial mass is a diagnostic dilemma considering its rarity. Other etiologies of an endobronchial mass include malignancy, TB, fungal infections (aspergillosis, histoplasmosis, blastomycosis), granulomatous disorders such a granulomatosis with polyangitis (GPA), amyloidosis and benign tumors such a lipoma and papilloma. Accurate diagnosis relies on histopathological examination and microbiology of the biopsy specimen and clinical correlation [13]. This atypical manifestation of sarcoidosis most commonly mimics lung malignancy, especially when accompanied by mediastinal lymphadenopathy. The presence of non-caseating granuloma alone does not definitively exclude malignancy as such granulomas have been documented in association with malignancies like small-cell lung cancer [14]. Therefore, tissue sampling from at least two non-contiguous sites is recommended in atypical presentations [15].

First line treatment with systemic glucocorticoids is the cornerstone of treatment for sarcoidosis. Approximately 30% of the patients may experience relapses following cessation/tapering of glucocorticoids; addition of disease modifying agents such as methotrexate/azathioprine are recommended. The response of endobronchial mass lesion to medical management is uncertain [12]. Corsello et al. documented complete resolution of an endobronchial mass following glucocorticoid therapy [16]. The reported cases in Table 1 illustrate the clinical patterns of endobronchial mass in sarcoidosis. Similar to index case report, endobronchial mass in sarcoidosis is more frequently observed in fourth-fifth decade of life and male predominance. Notably, most of the published case reports have described single mass lesion with right lower lobe being the most common site of involvement.

**Table 1:** Summary of case reports of sarcoidosis with endobronchial mass lesion

Case Report	Symptoms	CT findings	Bronchoscopy	Management and follow-up
Corsello et al. [16] 37-year-old male	Fever and cough for 1 month	Chest X-ray showed right lower lobe infiltrates	Endobronchial mass partially occluding apical basal segment of right lower lobe	Prednisolone 60 mg once daily. Symptomatic improvement and radiological resolution at one month follow up. Bronchoscopy showed complete resolution of endobronchial mass at two months follow-up.
Gonzalez et al. [17] 33-year-old male	Fever, cough and chest pain for 15 days	Mediastinal and right hilar lymphadenopathy	Right lower lobe friable mass	No treatment given. Asymptomatic at one year.
Thomas et al. [18] 45-year-old male	Progressive breathlessness and polyarthritis for two months	Bilateral hilar and mediastinal lymphadenopathy	Smooth polypoid lustrous mass occluding the medial segment of right middle lobe	Oral prednisolone. Symptomatic improvement at follow-up.
Rai et al. [9] 50-year-old male	Fever, cough and dyspnea; small joint pain	Bilateral hilar lymphadenopathy	Polypoid ball-like mass in right lower lobe bronchus occluding posterior basal segment	Oral prednisolone (0.5 mg/kg). Symptomatic improvement as follow
Orhun et al. [12] 42-year-old male	Progressive breathlessness and cough for 4 months	Interstitial reticular thickening; mediastinal and hilar lymphadenopathy	Endobronchial polyp in left upper lobe, left lower lobe and right lower lobe	Oral prednisolone
Present case 34-year-old male	Progressive shortness of breath and non-productive cough for 1 month	Mediastinal lymphadenopathy; multiple small peripheral lung nodules with mild right-sided pleural effusion	Polypoid mass lesion with a broad base in segmental bronchus of right lower lobe	Oral prednisolone (0.5 mg/kg) once daily

## Conclusions

This case report highlights an uncommon etiology of endobronchial mass emphasizing the importance of maintaining a broad differential diagnosis and requirement of comprehensive diagnostic evaluation. The uncommon manifestation of sarcoidosis as an endobronchial mass lesion should be recognized by primary care providers. To distinguish granulomatous illness from cancer, a biopsy from a large lesion is crucial. For the excision of an endobronchial mass, electrosurgical snaring combined with cryoextraction is the suitable choice.
